# Diagnostic and clinical experience of patients with pantothenate kinase-associated neurodegeneration

**DOI:** 10.1186/s13023-019-1142-1

**Published:** 2019-07-12

**Authors:** Randall D. Marshall, Abigail Collins, Maria L. Escolar, H. A. Jinnah, Thomas Klopstock, Michael C. Kruer, Aleksandar Videnovic, Amy Robichaux-Viehoever, Colleen Burns, Laura L. Swett, Dennis A. Revicki, Randall H. Bender, William R. Lenderking

**Affiliations:** 1 Formerly Retrophin, Inc., San Diego, CA USA; 2 Departments of Pediatrics and Neurology, University of Colorado, School of Medicine, Denver, CO USA; 3 Department of Pediatrics, University of Pittsburgh School of Medicine, Pittsburgh, PA USA; 4 Departments of Neurology and Human Genetics, Emory University School of Medicine, Atlanta, GA USA; 5 Department of Neurology, Friedrich-Baur-Institute, University of Munich, Munich, Germany; 6 German Center for Neurodegenerative Diseases (DZNE), Munich, Germany; 7 Munich Cluster for Systems Neurology (SyNergy), Munich, Germany; 8 Barrow Neurological Institute, Phoenix Children’s Hospital, University of Arizona College of Medicine, Phoenix, AZ USA; 9 Department of Neurology, Massachusetts General Hospital/Harvard Medical School, Boston, MA USA; 10 Department of Neurology, Washington University School of Medicine, St. Louis, MO USA; 11 Retrophin, Inc., 3721 Valley Centre Drive, Suite 200, San Diego, CA 92130 USA; 12 Evidera, Inc, Bethesda, MD USA

**Keywords:** PKAN, Burden of illness, Healthcare utilization, PKAN-ADL scale, Caregiver

## Abstract

**Background:**

Pantothenate kinase-associated neurodegeneration (PKAN) is an autosomal recessive neurodegenerative disorder with brain iron accumulation (NBIA).

**Objectives:**

To assess PKAN diagnostic pathway, history, and burden across the spectrum of PKAN severity from patient and/or caregiver perspectives.

**Methods:**

Caregivers of patients (*n* = 37) and patients themselves (*n* = 2) were interviewed in a validation study of the PKAN-Activities of Daily Living (ADL) scale. The current study used quartiles of the PKAN-ADL total score to divide patients by severity of impairment (Lowest, Second Lowest, Third Lowest, Highest). Diagnostic and treatment history, healthcare utilization, disease burden, and caregiver experience were compared between groups.

**Results:**

The analyses included data from 39 patients. Mean age at PKAN symptom onset (*P* = 0.0007), initial MRI (*P* = 0.0150), and genetic testing (*P* = 0.0016) generally decreased across the PKAN severity spectrum. The mean duration of illness did not differ among PKAN severity groups (range, 9.7–15.2 years; *P* = 0.3029). First MRI led to diagnosis in 56.4% of patients (range, 30.0–90.0%). A mean (SD) of 13.0 (13.1) medical and 55.2 (78.5) therapy visits (eg, physical, speech) occurred in the past year. More patients in the higher PKAN severity groups experienced multiple current functional losses and/or earlier onset of problems (*P*-values < 0.0500). Over half (56.8%) of caregivers experienced a change in employment because of caregiving. The percentage of patients requiring full-time caregiving increased across the PKAN severity spectrum (range, 11.1–100%; *P* = 0.0021).

**Conclusions:**

PKAN diagnosis was often delayed, most probably due to low awareness. Considerable burden of functional impairment and high healthcare utilization were found across the PKAN severity spectrum.

**Electronic supplementary material:**

The online version of this article (10.1186/s13023-019-1142-1) contains supplementary material, which is available to authorized users.

## Introduction

Pantothenate kinase-associated neurodegeneration (PKAN) is an autosomal recessive disorder due to mutations in the *PANK2* gene, with an estimated prevalence of one to two per million persons worldwide, and a highly variable phenotype [1–7]. PKAN is the most common disorder within the group of neurodegeneration with brain iron accumulation (NBIA) disorders [8]. Motor manifestations of PKAN include dystonia, chorea, pyramidal signs, parkinsonism, spasticity, dysarthria/anarthria, and dysphagia. Cognitive impairment, psychiatric features, oculomotor deficits, and retinopathy may also be present. The genotype/phenotype association is not well understood, and key features such as rate of progression, age of onset, and signs and symptoms are highly variable, even among siblings and individuals with identical mutations [9].

Because of its rarity, current knowledge about PKAN is based on case reports, case series, and personal observations presented by clinicians from major medical centers. This study was conducted to capture key features of the disease history and its progression from the patient and/or caregiver’s perspective, in a research sample of 39 patients and their primary caregivers. This relatively large patient sample for a PKAN study provides a good overview of the natural disease history of patients with PKAN, including presenting symptoms, methods of diagnosis, clinical history, and current clinical status. Data were collected in the context of a larger study aiming to develop the first PKAN-specific clinical outcomes assessment scale, the PKAN-Activities of Daily Living (PKAN-ADL) Scale [10].

## Methods

The evaluation of the PKAN-ADL was conducted in accordance with the Declaration of Helsinki, local independent ethics committee/institutional review board requirements, and good clinical practice guidelines. The study protocol received approval by Ethical and Independent Review Services, a central IRB. Written informed consent or assent was obtained from all patients and caregivers.

Study participants were recruited via the NBIA Disorders Association (NBIA-DA), clinicians treating PKAN patients, family networks, and social media. Recruitment continued until the pre-specified study enrollment target was met (*n* = 40). The full inclusion and exclusion criteria for caregivers and patients are previously published [10]. Briefly, the study recruited caregivers ≥18 years old of patients with a genetically confirmed diagnosis of PKAN who were at least 6 years old. Patients who were interviewed had to be at least 16 years old and able to speak clearly enough over the telephone to be interviewed by a stranger. Because difficulty with speech is common in patients with PKAN, verbal interviews are often difficult or impossible. For this reason, we recruited patients with PKAN and their primary caregivers. We interviewed the primary caregiver of a patient with PKAN rather than the patient when PKAN symptoms precluded the possibility of an interview, or if the patient was younger than 16 years old. Patients or caregivers with any clinically relevant physical or mental conditions that would interfere with study participation were excluded.

The measurement evaluation study of the PKAN-ADL consisted of semi-structured interviews via telephone with caregivers and patients. The interview contained a structured evaluation of the PKAN-ADL in addition to an open-ended section about PKAN-related medical history and symptoms (Additional file 1: Table S1). The interviewer administered a patient and/or caregiver sociodemographic information form, a disease-specific history form, and a series of questionnaires to the caregiver and/or patient as appropriate. The interviewer recorded patient and caregiver responses and the audio of the interviews was recorded. Data collection interviews lasted 90–120 min. For the follow-up interview, participants completed the 12-item PKAN-ADL, requiring approximately 20 min, roughly 2 weeks following the initial interview.

### Study measures

#### Sociodemographic questionnaires

The sociodemographic questionnaire for the caregivers and patients included items on age, sex, race/ethnicity, and employment status.

#### PKAN-specific medical history form

The PKAN-specific medical history form collected clinical information about the patient’s experience with PKAN, including age of onset, duration of illness, method of diagnosis (MRI versus genetic or clinical), standardized questions on milestones of disease progression, and treatments received for PKAN.

#### PKAN-ADL

The PKAN-ADL assesses 12 domains of activities of daily living: speech; drooling; swallowing; writing; eating tasks; dressing; personal hygiene tasks; turning or changing position in bed; sitting; falling; walking; and discomfort or pain [10]. Higher scores indicate higher levels of severity of impairment. The five-point scale response options for the PKAN-ADL range from 0 (indicating no problems) to 4 (indicating inability to perform the activity). Quartiles of the scale total summary score were used to define four severity of impairment groups across the spectrum of PKAN severity: Lowest (score < 15), Second Lowest (≥15 to < 25), Third Lowest (≥25 to < 38), and Highest (≥38).

#### Other study measures

Several validated questionnaires were administered within the measurement study to evaluate convergent and divergent validity, as previously published [10]. The measures included selected Quality of Life in Neurological Disorders measures [11], selected attributes of the Health Utilities Index Mark 3 [12], and the Stroke Aphasic Depression Questionnaire [13].

### Data analysis

The data analyses explored descriptive statistics (N, mean, SD, median, minimum, maximum, and floor and ceiling effects) of the PKAN-specific medical history form and the sociodemographic forms for the patients and caregivers. To identify patient groups across the spectrum of PKAN disease severity, since there is no established and validated measure of disease severity or severity-based disease classification to date, we used quartiles of the PKAN-ADL total score to define four severity of impairment groups, as described above. Severity of impairment group differences were tested using F tests and chi-square tests.

### Qualitative data analysis

Qualitative analyses in the study followed the principles of thematic narrative analysis [14] with the goal of identifying themes associated with patient and caregiver feedback regarding the pathway to diagnosis of PKAN, healthcare utilization, and current symptoms and functioning.

## Results

### Sociodemographic characteristics of patients and caregivers

The sample was composed of 37 primary caregivers of patients with PKAN (*n* = 37 patients) and 2 patients with PKAN, for a total of 39 participants providing data on 39 patients (Table 1). Caregivers and patients resided in the United States (US) (*n* = 35), Canada (n = 3), and Germany (*n* = 1). The median age (range) of the caregivers was 45.8 (24.1 to 67.6) years, caregivers were mostly female (26/37; 70.3%), and caregivers were primarily parents of the PKAN patients (33/37; 89.2%). Most caregivers were related to the patient, lived with the patient, and were employed full-time or part-time (Table 1). Patients (*n* = 39) had a median (range) age of 19.1 (6.4 to 42.6) years, and were mostly White (35/39; 89.7%), male (24/39; 61.5%), and did not live alone (37/39; 94.9%). A large proportion of patients were students (19/39; 48.7%); others were employed, unemployed, or disabled (Table 1). Using the quartiles on the PKAN-ADL total score to define four PKAN severity of impairment groups, 9 patients were placed in the Lowest group, 10 in the Second Lowest group, 10 in the Third Lowest group, and 10 in the Highest severity of impairment group. There was a trend toward a difference in age at study enrollment across the PKAN disease spectrum (*P* = 0.0739) (Table 1). The percentages of patients living with someone other than parents or siblings were significantly different across PKAN severity of impairment groups (*P* = 0.0222). No other sociodemographic characteristics significantly differed across PKAN severity groups.

**Table 1:** Sociodemographic characteristics

Characteristic	Caregivers (*N* = 37)	All patients (*N* = 39)	Lowest (*n* = 9)	Second Lowest (*n* = 10)	Third Lowest (*n* = 10)	Highest (*n* = 10)	*P*-value^a^
Age, y	*n* = 35/37	*n* = 38/39			*n* = 9/10		0.0739
Mean (SD)	46.8 (12.5)	20.2 (8.4)	24.7 (5.8)	19.1 (7.1)	22.5 (11.0)	15.4 (6.9)	
Median	45.8	19.1	22.8	19.1	23.1	14.2	
Range (min, max)	(24.1–67.6)	(6.4–42.6)	(17.0–33.2)	(6.4–30.0)	(7.9–42.6)	(7.0–29.8)	
Gender, n (%)							0.4626
Male	11 (29.7%)	24 (61.5%)	4 (44.4%)	8 (80.0%)	6 (60.0%)	6 (60.0%)	
Female	26 (70.3%)	15 (38.5%)	5 (55.6%)	2 (20.0%)	4 (40.0%)	4 (40.0%)	
With whom patient lives, n (%)							
Parents/Siblings	NA	33 (84.6%)	8 (88.9%)	9 (90.0%)	6 (60.0%)	10 (100%)	0.0222
Spouse/Partner	NA	0 (0.0%)	0 (0.0%)	0 (0.0%)	0 (0.0%)	0 (0.0%)	
Other family member	NA	0 (0.0%)	0 (0.0%)	0 (0.0%)	0 (0.0%)	0 (0.0%)	
Alone	NA	2 (5.1%)	1 (11.1%)	1 (10.0%)	0 (0.0%)	0 (0.0%)	
Other	NA	4 (10.3%)	0 (0.0%)	0 (0.0%)	4 (40.0%)	0 (0.0%)	
Relationship to the patient, n (%)							
Parent	33 (89.2%)	NA					
Grandparent	0 (0.0%)	NA					
Other relative	1 (2.7%)	NA					
Professional caregiver	1 (2.7%)	NA					
Other	2 (5.4%)	NA					
Caregiver lives with patient, n (%)							
Yes	31 (83.8%)	NA					
No	6 (16.2%)	NA					
Numbers of hours per week spent with patient	*n* = 6/37						
Mean (SD)	16.0 (16.7)	NA					
Median	11.0	NA					
Range (min, max)	(1.0–36.0)	NA					
Ethnic background, n (%)							0.4810
Hispanic or Latino	4 (10.8%)	6 (15.4%)	2 (22.2%)	2 (20.0%)	0 (0.0%)	2 (20.0%)	
Not Hispanic or Latino	33 (89.2%)	33 (84.6%)	7 (77.8%)	8 (80.0%)	10 (100%)	8 (80.0%)	
Racial background, n (%)		*n* = 38/39		*n* = 9/10			0.5207
White	33 (89.2%)	35 (89.7%)	8 (88.9%)	9 (90.0%)	9 (90.0%)	9 (90.0%)	
Black or African American	0 (0.0%)	0 (0.0%)	0 (0.0%)	0 (0.0%)	0 (0.0%)	0 (0.0%)	
Asian	3 (8.1%)	2 (5.1%)	0 (0.0%)	0 (0.0%)	1 (10.0%)	1 (10.0%)	
Native Hawaiian or other Pacific Islander	1 (2.7%)	1 (2.6%)	1 (11.1%)	0 (0.0%)	0 (0.0%)	0 (0.0%)	
Employment status, n (%)							0.4198
Employed, full-time or part-time	28 (75.7%)	4 (10.3%)	3 (33.3%)	1 (10.0%)	0 (0.0%)	0 (0.0%)	
Homemaker	4 (10.8%)	0 (0.0%)	0 (0.0%)	0 (0.0%)	0 (0.0%)	0 (0.0%)	
Student	0 (0.0%)	19 (48.7%)	2 (22.2%)	6 (60.0%)	4 (40.0%)	7 (70.0%)	
Unemployed	0 (0.0%)	4 (10.3%)	1 (11.1%)	0 (0.0%)	2 (20.0%)	1 (10.0%)	
Retired	4 (10.8%)	0 (0.0%)	0 (0.0%)	0 (0.0%)	0 (0.0%)	0 (0.0%)	
Disabled	1 (2.7%)	12 (30.8%)	3 (33.3%)	3 (30.0%)	4 (40.0%)	2 (20.0%)	
Highest educational level, n (%)^b^							0.1213
Elementary/primary school	0 (0.0%)	14 (35.9%)	0 (0.0%)	3 (30.0%)	5 (50.0%)	6 (60.0%)	
Secondary/high school	7 (18.9%)	13 (33.3%)	2 (22.2%)	5 (50.0%)	3 (30.0%)	3 (30.0%)	
Some college	9 (24.3%)	9 (23.1%)	5 (55.6%)	2 (20.0%)	1 (10.0%)	1 (10.0%)	
College degree	7 (18.9%)	2 (5.1%)	1 (11.1%)	0 (0.0%)	1 (10.0%)	0 (0.0%)	
Some graduate school	4 (10.8%)	0 (0.0%)	0 (0.0%)	0 (0.0%)	0 (0.0%)	0 (0.0%)	
Postgraduate degree	7 (18.9%)	0 (0.0%)	0 (0.0%)	0 (0.0%)	0 (0.0%)	0 (0.0%)	
Technical or vocational degree	3 (8.1%)	0 (0.0%)	0 (0.0%)	0 (0.0%)	0 (0.0%)	0 (0.0%)	
Other	0 (0.0%)	1 (2.6%)	1 (11.1%)	0 (0.0%)	0 (0.0%)	0 (0.0%)	
Educational achievement limited by PKAN, n (%)		*n* = 38/39			*n* = 9/10		0.1871
Yes	NA	30 (76.9%)	5 (55.6%)	8 (80.0%)	7 (70.0%)	10 (100%)	
No	NA	8 (20.5%)	4 (44.4%)	2 (20.0%)	2 (20.0%)	0 (0.0%)	
Change in employment status due to caregiving, n (%)^c^							0.0128
Yes	21 (56.8%)	NA	5 (71.4%)	9 (90.0%)	2 (20.0%)	5 (50.0%)	
No	16 (43.2%)	NA	2 (28.6%)	1 (10.0%)	8 (80.0%)	5 (50.0%)	

^a^Comparisons between quartile groups based on F tests for continuous data and chi-square tests for categorical data; ^b^Responses are not mutually exclusive; ^c^Employment status change for caregivers is shown by PKAN severity of impairment patient quartile groups

NA, not applicable; PKAN, pantothenate kinase-associated neurodegeneration; SD, standard deviation

### PKAN clinical history and pathway to diagnosis

#### Presenting symptoms of PKAN

The most common first symptoms or functional limitations noted in patients (often not limited to one symptom) were difficulties with walking, speech, and writing, followed by several other less common initial signs or symptoms such as dystonia or emotional and behavioral problems (Table 2). The percentages of patients presenting with difficulty walking were significantly different (*P* = 0.0127), showing an increase across the spectrum of PKAN severity. The first PKAN symptom (ie, the presenting symptom, which varied among patients) led to the first doctor visit in 56.4% (22/39) of patients, ranging from 50% of patients in the Second Lowest (5/10) and the Highest (5/10) groups to 70% of patients in the Third Lowest (7/10) group.

**Table 2:** PKAN clinical history and pathway to diagnosis

Characteristic	All patients (*N* = 39)	Lowest (*n* = 9)	Second Lowest (*n* = 10)	Third Lowest (*n* = 10)	Highest (*n* = 10)	*P*-value^a^
Presenting symptoms of PKAN, n (%)^b^						
Walking difficulty	27 (69.2%)	3 (33.3%)	6 (60.0%)	8 (80.0%)	10 (100%)	0.0127
Speech	12 (30.8%)	1 (11.1%)	5 (50.0%)	2 (20.0%)	4 (40.0%)	0.2296
Swallowing	2 (5.1%)	1 (11.1%)	1 (10.0%)	0 (0.0%)	0 (0.0%)	0.5259
Writing	9 (23.1%)	4 (44.4%)	3 (30.0%)	0 (0.0%)	2 (20.0%)	0.1306
Emotional/behavioral problems	6 (15.4%)	3 (33.3%)	1 (10.0%)	1 (10.0%)	1 (10.0%)	0.4080
Other problems	19 (48.7%)	4 (44.4%)	6 (60.0%)	6 (60.0%)	3 (30.0%)	0.4776
Dystonia at PKAN presentation, n (%)^b^	6 (15.4%)	2 (22.2%)	2 (20.0%)	1 (10.0%)	1 (10.0%)	0.8176
Dystonia: mouth/tongue	4 (10.3%)	2 (22.2%)	1 (10.0%)	0 (0.0%)	1 (10.0%)	
Dystonia: neck	1 (2.6%)	0 (0.0%)	0 (0.0%)	0 (0.0%)	1 (10.0%)	
Dystonia: hand	6 (15.4%)	2 (22.2%)	2 (20.0%)	1 (10.0%)	1 (10.0%)	
Dystonia: foot	2 (5.1%)	1 (11.1%)	0 (0.0%)	0 (0.0%)	1 (10.0%)	
Dystonia: back/trunk	3 (7.7%)	1 (11.1%)	1 (10.0%)	0 (0.0%)	1 (10.0%)	
Dystonia: other	2 (5.1%)	1 (11.1%)	0 (0.0%)	0 (0.0%)	1 (10.0%)	
Age at onset, y	*n* = 38/39			*n* = 9/10		0.0007
Mean (SD)	8.0 (5.8)	12.7 (4.3)	9.4 (5.5)	7.3 (5.2)	2.9 (3.8)	
Median	7.0	14.0	10.0	7.0	1.0	
Range (min, max)	(< 1.0–20.0)	(7.0–20.0)	(1.0–18.0)	(1.0–16.0)	(< 1.0–12.0)	
Problem leading to first doctor visit, n (%)	*n* = 36/39	*n* = 8/9	*n* = 9/10	*n* = 9/10		
Same as “first symptom”	22 (56.4%)	5 (55.6%)	5 (50.0%)	7 (70.0%)	5 (50.0%)	
Other	14 (35.9%)	3 (33.3%)	4 (40.0%)	2 (20.0%)	5 (50.0%)	
MRI, n (%)						0.3953
Yes	38 (97.4%)	9 (100%)	10 (100%)	10 (100%)	9 (90.0%)	
No	1 (2.6%)	0 (0.0%)	0 (0.0%)	0 (0.0%)	1 (10.0%)	
Age at first MRI, y	*n* = 36/38	*n* = 8/9		*n* = 9/10		0.0150
Mean (SD)	10.4 (6.7)	15.0 (6.9)	11.5 (5.0)	10.8 (7.8)	5.4 (3.8)	
Median	10.0	16.0	10.0	10.0	4.5	
Range (min, max)	(1.0–26.0)	(5.0–25.0)	(5.0–23.0)	(1.0–26.0)	(1.0–12.0)	
Did first MRI diagnose PKAN?	*n* = 38/39			*n* = 9/10		0.1005
Yes	22 (56.4%)	5 (55.6%)	9 (90.0%)	5 (50.0%)	3 (30.0%)	
No	16 (41.0%)	4 (44.4%)	1 (10.0%)	4 (40.0%)	7 (70.0%)	
Genetic testing obtained						0.5259
Yes	37 (94.9%)	8 (88.9%)	9 (90.0%)	10 (100%)	10 (100%)	
No	2 (5.1%)	1 (11.1%)	1 (10.0%)	0 (0.0%)	0 (0.0%)	
Age genetic testing led to PKAN diagnosis	*n* = 36/39	*n* = 8/9	*n* = 9/10	*n* = 9/10		0.0116
Mean (SD)	12.1 (6.9)	17.4 (4.3)	11.9 (5.1)	13.2 (9.7)	7.2 (3.0)	
Median	10.5	16.0	10.0	13.0	7.0	
Range (min, max)	(4.0–36.0)	(12.0–26.0)	(5.0–23.0)	(4.0–36.0)	(4.0–12.0)	
Age MRI diagnosed PKAN if MRI diagnosis	*n* = 1/22	*n* = 1/5	*n* = 0/9	*n* = 0/5	*n* = 0/3	
Mean (SD)	11.0	11.0				
Median	11.0	11.0				
Range (min, max)	(11.0–11.0)	(11.0–11.0)				
Number of doctors seen prior to PKAN diagnosis						0.4213
Mean (SD)	4.6 (3.5)	5.2 (4.7)	3.1 (3.2)	4.5 (2.5)	5.6 (3.6)	
Median	4.0	3.0	2.0	4.0	5.5	
Range (min, max)	(1.0–15.0)	(1.0–15.0)	(1.0–10.0)	(1.0–10.0)	(1.0–12.0)	
Location of doctors consulted, n (%)^b^						
Community	16 (41.0%)	5 (55.6%)	3 (30.0%)	6 (60.0%)	2 (20.0%)	
Specialist center doctors	37 (94.9%)	7 (77.8%)	10 (100%)	10 (100%)	10 (100%)	

^a^Comparisons among quartile groups based on F tests for continuous data and chi-square tests for categorical data; ^b^Responses are not mutually exclusive; ^c^Patient sample sizes are based on the number of patients reporting first MRI led to diagnosis of PKAN

MRI, magnetic resonance imaging; PKAN, pantothenate kinase-associated neurodegeneration, SD, standard deviation

#### Age at onset

**Fig. 1:**
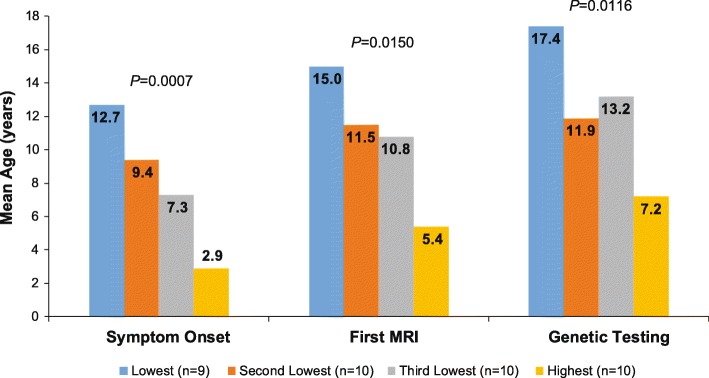
Mean age at onset of PKAN symptoms, first MRI, and genetic testing. MRI, magnetic resonance imaging; PKAN, pantothenate kinase-associated neurodegeneration

The median age at symptom onset was 7.0 years. Ages at onset were significantly different across PKAN severity groups (*P* = 0.0007), showing a decrease in mean ages across the disease spectrum (Fig. 1). There was a wide range in mean age at onset across the severity of impairment groups, from 7.0 to 20.0 years old (Lowest), 1.0 to 18.0 years old (Second Lowest), 1.0 to 16.0 years old (Third Lowest), and < 1.0 to 12.0 years old (Highest), consistent with known PKAN heterogeneity (Table 2). Mean (SD; median) years from symptom onset to PKAN diagnosis was not significantly different across PKAN severity groups (Lowest = 3.9 [4.4; 2.0], Second Lowest = 2.6 [3.1; 1.5], Third Lowest = 5.8 [9.2; 1.0], Highest = 4.3 [2.9; 3.5]; *P* = 0.6381).

#### First MRI and PKAN diagnosis

Most patients (38/39 [97.4%]) had an MRI and the first MRI occurred at a mean (SD) age of 10.4 (6.7) years. Ages at first MRI were significantly different across PKAN severity groups (*P* = 0.0150), generally showing a decrease in mean ages across the disease spectrum (Fig. 1). First MRI led to a diagnosis in just over half of the patients (22/39 [56.4%]), with no significant difference across PKAN severity groups (Table 2).

#### Genetic testing and PKAN diagnosis

Genetic testing was obtained by most patients (37/39 [94.9%]), leading to a PKAN diagnosis on average at age 12.1 (6.9) years. Ages at diagnosis based on genetic testing were significantly different across PKAN severity groups (*P* = 0.0116), generally showing a decrease in mean ages across the disease spectrum (Fig. 1). Mean (SD; median) years from symptom onset to diagnosis based on genetic testing was not significantly different across PKAN severity groups (Lowest = 4.5 [4.4; 3.5], Second Lowest = 3.1 [3.1; 4.0], Third Lowest = 5.9 [9.3; 1.0], Highest = 4.3 [2.9; 3.5]; *P* = 0.7584).

#### Physician visits to obtain diagnosis

The patient journey to diagnosis involved a mean (SD; median) of 4.6 (3.5; 4.0) different physician consultations prior to diagnosis, with a range of 1.0 to 15.0 (Table 2). By PKAN severity, the number of physician consultations prior to diagnosis did not significantly differ across groups (*P* = 0.4213), and ranged from a mean (SD; median) of 3.1 (3.2: 2.0) in the Second Lowest group to 5.6 (3.6; 5.5) in the Highest group. Eventually, nearly all patients were referred to or initiated contact with specialist centers (37/39, 94.9%). One caregiver reported consulting 3 pediatricians, 4 neurologists, and a geneticist outside of the community, prior to obtaining a diagnosis, and driving 3–5 h round trip per doctor visit. She reported that over a span of 7 years of appointments, the doctors “knew something was wrong” with her child, but were unable to make the diagnosis.

### Healthcare utilization

#### Medical care

The percentages of patients who received various types of treatments (medication, vitamins or over-the-counter treatments, injections, therapies such as physical, speech, or occupational) were similar across PKAN severity groups (Table 3). The percentages of patients with surgery other than deep brain stimulation device placement were significantly different across PKAN severity groups (*P* = 0.0006), being more common in the Highest severity group. Across all PKAN patients, a mean (SD; median) of 13.0 (13.1; 8.0) medical visits and 55.2 (78.5; 22.5) therapy visits occurred in the past year. Number of medical or therapy visits did not significantly differ across PKAN severity groups (*P*-values > 0.2187) (Table 3).

**Table 3:** Healthcare utilization

	All patients *N* = 39	Lowest *n* = 9	Second Lowest *n* = 10	Third Lowest *n* = 10	Highest *n* = 10	*P*-value
PKAN treatments received						
Medication from doctor, n (%)	39 (100%)	9 (100%)	10 (100%)	10 (100%)	10 (100%)	
Vitamins or OTC, n (%)	35 (89.7%)	8 (88.9%)	7 (70.0%)	10 (100%)	10 (100%)	0.0886
Injections (botulinum toxin, etc), n (%)	25 (64.1%)	4 (44.4%)	7 (70.0%)	5 (50.0%)	9 (90.0%)	0.1422
Therapies (physical, speech, occupational, etc), n (%)	36 (92.3%)	7 (77.8%)	10 (100%)	9 (90.0%)	10 (100%)	0.2198
Surgery, n (%)	16 (41.0%)	2 (22.2%)	2 (20.0%)	3 (30.0%)	9 (90.0%)	0.0036
DBS device	9 (23.1%)	2 (22.2%)	2 (20.0%)	2 (20.0%)	3 (30.0%)	0.9443
Other surgery	14 (35.9%)	2 (22.2%)	1 (10.0%)	2 (20.0%)	9 (90.0%)	0.0006
Other treatment, n (%)^a^	14 (35.9%)	1 (11.1%)	5 (50.0%)	4 (40.0%)	4 (40.0%)	0.3322
PKAN healthcare utilization past year						
Medical visits						0.3446
Mean (SD)	13.0 (13.1)	12.2 (16.8)	7.6 (5.3)	13.9 (15.6)	18.3 (11.9)	
Median	8.0	6.0	5.0	7.0	15.0	
Range (min, max)	(1.0–56.0)	(2.0–56.0)	(1.0–15.0)	(3.0–50.0)	(1.0–40.0)	
Therapy visits	*n* = 38/39			*n* = 9/10		0.2188
Mean (SD)	55.2 (78.5)	23.1 (50.2)	33.7 (32.1)	85.1 (126.5)	78.7 (69.1)	
Median	22.5	6.0	26.5	20.0	46.0	
Range (min, max)	(0.0–384.0)	(0.0–156.0)	(0.0–100.0)	(0.0–384.0)	(1.0–208.0)	
Caregiving required						
Part-time caregiver, n (%)	25 (64.1%)	3 (33.3%)	6 (60.0%)	8 (80.0%)	8 (80.0%)	0.1656
Age at onset, y	*n* = 23/25	*n* = 1/3				
Mean (SD)	11.5 (7.6)	12.0 (NA)	17.7 (8.0)	10.8 (7.9)	7.6 (4.8)	0.0975
Median	11.0	12.0	19.0	11.0	7.0	
Range (min, max)	(< 1.0–28.0)	(12.0–12.0)	(6.0–28.0)	(< 1.0–20.0)	(< 1.0–14.0)	
Full-time caregiver, n (%)	21 (53.8%)	1 (11.1%)	3 (30.0%)	7 (70.0%)	10 (100%)	0.0021
Age at onset, y	*n* = 20/21		*n* = 2/3			
Mean (SD)	10.3 (6.6)	19.0 (NA)	16.5 (9.2)	9.7 (7.3)	8.5 (5.1)	0.2379
Median	10.0	19.0	16.5	12.0	8.5	
Range (min, max)	(< 1.0–23.0)	(19.0–19.0)	(10.0–23.0)	(< 1.0–20.0)	(< 1.0–17.0)	

^a^Examples of other treatments for PKAN included leg braces, hippotherapy (horseback riding), and participating in a clinical trial (eg, deferiprone)

*DBS* deep brain stimulation; *OTC* over the counter; *PKAN* pantothenate kinase-associated neurodegeneration; *SD* standard deviation

#### Caregiving

Over half of the patients (21/39, 53.8%) required a full-time caregiver (Table 3). The percentages of patients who required full-time caregiving were significantly different across PKAN severity groups (*P* = 0.0021), showing an increase across the disease spectrum. Median age at onset requiring full-time caregiving ranged from 8.5 years old (Highest group) to 19.0 years old (Lowest group) (*P* = 0.2379), and median age at onset requiring part-time caregiving ranged from 7.0 years old (Highest group) to 19.0 years old (Second Lowest group) (*P* = 0.0975). Reasons given for the need for a full-time caregiver included progression of dementia, unable to be left alone due to both developmental issues and problems with falling, and loss of mobility. Some parents reported sharing caregiving duties with a professional caregiver. Examples of parental caregiving duties included assistance with transportation, aiding patient when patient’s hand “tires out” or hand contractions interfere with eating/dressing, assistance with personal hygiene and medications, and monitoring to prevent the patient from wandering off.

Over half (21/37, 56.8%) of caregivers experienced a change in employment status because of caregiving. The percentages of caregivers who experienced a change in employment status were significantly different across PKAN severity groups (*P* = 0.0128), ranging from 20.0% (2/10, Third Lowest group) to 90.0% (9/10, Second Lowest group). One parent reported that he retired early to provide full-time care.

### Current symptoms and functioning

The PKAN severity of impairment groups did not significantly differ in mean (SD) years since onset of symptoms (Lowest = 12.0 [5.5], Second Lowest = 9.7 [3.7], Third Lowest = 15.2 [9.3], Highest = 12.5 [5.2] years; *P* = 0.3029) or mean (SD) years since diagnosis (Lowest = 8.1 [4.6], Second Lowest = 7.1 [4.6], Third Lowest = 9.4 [5.8], Highest = 8.2 [4.9] years; *P* = 0.8071).

#### Problems with walking

**Fig. 2:**
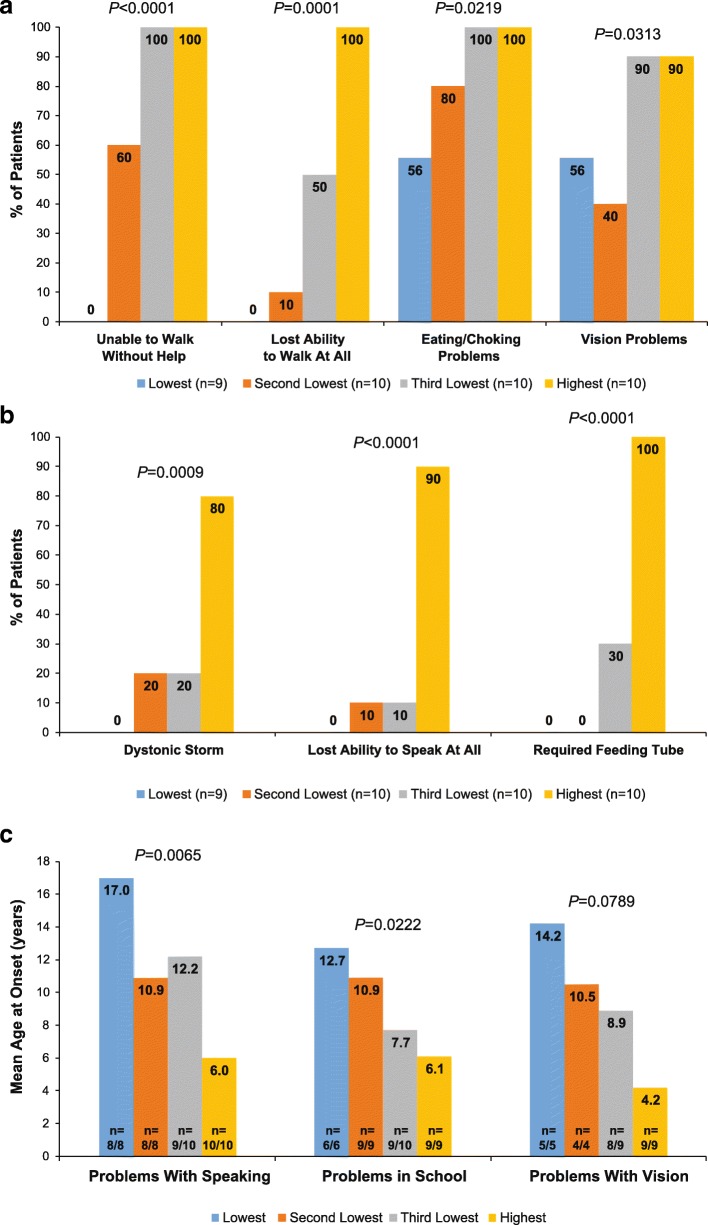
PKAN symptoms and functional limitations with increased frequency across the spectrum of PKAN severity (**a**), Greater frequency in the Highest Severity group (**b**), or younger age at onset across PKAN severity (**c**). In panel C, the n values show the actual number of patients reporting age at onset (numerator) and the total number of patients who experienced the problem (denominator) within each severity group. PKAN, pantothenate kinase-associated neurodegeneration

Most patients experienced problems with walking (26/39, 66.7%). The percentages of patients unable to walk without help and unable to walk at all were significantly different across PKAN severity groups (*P*-values ≤0.0001), generally showing an increase across the disease spectrum (Fig. 2a). Age at onset did not significantly differ for unable to walk without help (*P* = 0.1447), ranging from a mean (SD) age of 7.7 (4.2) years (Highest group) to 13.6 (8.6) years (Second Lowest group), or unable to walk at all (*P* = 0.3878), ranging from 10.4 (7.4) years (Highest group) to 18.0 years in the single patient in the Second Lowest group.

#### Problems with speaking

Most patients (36/39 [92.3%]) had trouble with speaking or being understood. There was no significant difference across PKAN severity groups (*P* = 0.2671), ranging from 80.0% (8/10 Second Lowest group) to 100.0% (10/10 each in the Third Lowest and Highest groups). The percentages of patients with lost ability to speak at all were significantly different across PKAN severity groups (*P* < 0.0001), being more common in the Highest severity group (Fig. 2b). Ages at onset for problems with speaking were significantly different across PKAN severity groups (*P* = 0.0065); mean ages generally decreased across the disease spectrum (Fig. 2c).

#### Problems with eating

The percentages of patients with eating or choking problems were significantly different across PKAN severity groups (*P* = 0.0219), generally showing an increase across the disease spectrum (Fig. 2a). Age at onset did not significantly differ across PKAN severity groups (*P* = 0.1064), ranging from a mean (SD) age of 9.4 (5.1) years (Highest group) to 18.0 (7.5) years (Lowest group). The need for placement of a feeding tube was significantly different across PKAN severity groups (*P* < 0.0001) and was generally more common in the Highest severity group (Fig. 2b). Age at onset did not significantly differ across PKAN severity groups (*P* = 0.3626), ranging from a mean (SD) age of 10.9 (3.7) years (Highest group) to 14.5 (10.6) years (Third Lowest group), with no patients in the two lowest severity groups reporting requirement of a feeding tube.

#### Problems with vision and breathing

Most patients had problems with vision (27/39 [69.2%]). The percentages of patients with vision problems were significantly different across PKAN severity groups (*P* = 0.0313), generally showing an increase across the disease spectrum (Fig. 2a). There was a trend toward different ages at onset for problems in vision across PKAN severity groups (*P* = 0.0789, Fig. 2c). One patient each in the Third Lowest severity group and the Highest severity group required the placement of a tracheostomy tube.

#### Dystonic storms

The percentages of patients who had ever experienced a dystonic storm were significantly different across PKAN severity groups (*P* = 0.0009), being more common in the Highest severity group (Fig. 2b). There was no significant difference in age at onset across PKAN severity groups (*P* = 0.3735), ranging from a mean (SD) age of 11.0 (5.7) years (Third Lowest group) to 20.0 (4.2) years (Second Lowest group).

#### Problems in school of any kind related to PKAN

Most patients (34/39, 87.2%) had problems in school. There was no significant difference in school problems across PKAN severity groups (*P* = 0.1717), ranging from 66.7% (6/9 Lowest group) to 100% (10/10 Third Lowest group). Ages at onset for school problems were significantly different across PKAN severity groups (*P* = 0.0222), showing a decrease in mean age across the disease spectrum (Fig. 2c).

#### Thematic analyses of burden of illness

Thematic analyses of spontaneous statements during the patient and caregiver interviews were used to identify key areas of illness burden for patients. Caregivers mentioned patients’ struggles to adapt to multiple losses of functioning (mobility, speech, ability to eat, visual impairment), loss of privacy due to the need for a caregiver, and social isolation because of loss of mobility, educational difficulties, difficulties communicating, and being moved out of their social group to special education programs. Examples of speech problems spontaneously reported by caregivers and patients include mumbling or words being garbled. Examples of reported school problems include the need for a modified program or for a full-time personal aide or nurse in the classroom, emotional and behavioral problems, and problems with handwriting or speech. Additionally, some caregivers spontaneously mentioned anger outbursts and mood swings in patients that they described as having difficulty with adjustment to the diagnosis. Painful episodes of dystonia and dystonic storms were described as adding to the patient burden.

## Discussion

These data present a clinically rich perspective on patients with PKAN across a range of functional impairment. Because these data were collected from caregiver and patient interviews, rather than physician records, they offer a unique perspective in relation to other similar size case series. Moreover, the 35 US patients may have represented 6.0–12.0% of the estimated US population with PKAN (US prevalence estimated as *n* = 318–636 [15]), and there was broad geographic representation.

There are several noteworthy findings. In general, severity of symptoms and problems, key medical history milestones, and burden of illness varied as would be expected with functional impairment, further supporting the validity of the PKAN-ADL [10]. Nearly 70.0% of families reported that the patient had problems with walking as a presenting symptom. This might include a range of neurological causes, including dystonia and parkinsonism. High-severity PKAN presented uniformly with walking difficulties, whereas heterogeneity of presentation for lower severity groups may contribute to delayed diagnosis. The median age of PKAN symptom onset of 7.0 years represented a wide range of ages (< 1.0–20.0 years). In nearly half the sample, the first symptom did not lead to a physician encounter, suggesting an insidious onset that only in retrospect is consistent with PKAN.

As with many rare diseases, a delay of up to several years prior to receiving an accurate diagnosis, over multiple physician visits, was the norm across the spectrum of PKAN severity. On average, the delay from symptom onset to diagnosis ranged from ~ 2.5 to 5.5 years, and the average delay from symptom onset to diagnosis based on genetic testing ranged from 3 to almost 6 years. Despite the “eye-of-the-tiger” sign on MRI being widely cited as near pathognomonic [8], only slightly more than half of the patients were diagnosed based on the first MRI. This finding suggests that the eye-of-the-tiger sign may not be as universally present as previously thought [16]. Alternatively, it could indicate the need for ongoing physician education to improve recognition of this radiological sign when it is present for PKAN diagnosis. It is also possible that for some of these patients, genetic testing may have confirmed the PKAN diagnosis prior to first MRI. Further study of the presenting features of PKAN in relation to disease severity and progression may inform earlier diagnosis and treatment.

These data reveal the extraordinary burden of illness borne by most families and patients across the spectrum of PKAN severity. Patients struggle with multiple losses as their functioning deteriorates through disease progression. Caregivers devote themselves part-time to full-time to assisting with daily activities, and transporting patients to a large number of medical visits, with an average of 13 physician visits and 55 therapy visits annually. Although economic status was not directly assessed, change in caregiver employment status implies a change in economic status. Finally, the combined costs of medical expenses (medical equipment and supplies, doctor’s visits, surgeries, therapy visits), expenses for paid caregiving, and transportation expenses almost certainly create substantial economic burden for caregivers.

Patients with PKAN across the spectrum of severity of functional impairment did not significantly differ in duration of illness (ranging from 9.7 years in the Second Lowest group to 15.2 years in the Third Lowest group), in contrast to the finding of Darling and colleagues (2017) [17] in a similarly sized cohort. This may reflect differences in the two cohorts, or in the scales themselves. Patients in the higher severity groups had younger age at onset for PKAN symptoms and functional impairments. The median age at symptom onset across the spectrum of PKAN severity ranged from 1.0 years old in the Highest severity group, to 7.0 and 10.0 years old in the Third Lowest and Second Lowest groups, respectively, to 14.0 years old in the Lowest group, but with broad overlapping ranges (< 1.0–12.0; 1.0–16.0; 1.0–18.0; and 7.0–20.0, respectively), making age at onset itself of little practical use for disease subtyping. The most parsimonious explanation for these data is that PKAN is best viewed as a phenotypic spectrum rather than a disease with distinct subtypes (“classic” versus “atypical”), similar to many other inherited disorders.

A limitation of this study is its relatively small sample size, which may limit generalizability, although this is also a limitation with other case series in PKAN (eg, Sachin et al. 2009 [5] [*n* = 16]; Tomic et al. 2015 [7] [*n* = 9]; Hayflick et al. 2003 [2] [*n* = 123]; and Hartig et al. 2006 [1] [*n* = 72]). This study’s design did not include physician ratings or records. However, no other published study to date reports a detailed analysis of the patient journey, and patient and caregiver burden, in relation to overall functional impairment, which is the objective of this report. Our cohort was primarily drawn from North America and there may be geographic differences in mutation distribution [18]. We recruited mostly caregivers into the study rather than patients, which reflects the clinical reality that difficulty with communication and cognitive disability are core functional impairments of this disease, making verbal interviews with patients difficult or impossible.

## Conclusions

These data derived from interviews with patients and caregivers help to further elucidate the diagnostic pathway and clinical experiences associated with PKAN. Across the spectrum of disease severity, PKAN is associated generally with a pre-adolescent age at onset, can be difficult to diagnose even with MRI, has a considerable burden of functional impairments, and has high healthcare utilization. Improved understanding of the real-world implications of PKAN across the spectrum of disease severity for patients and their caregivers can guide therapeutic planning and multi-functional medical team management.

## Additional file

Additional file 1: **Table S1.** PKAN-specific medical history form. (DOCX 18 kb)

## Data Availability

The data that support the findings of this study are available from the corresponding author on request.
