# Supplementary material for: Diagnostic and clinical experience of patients with pantothenate kinase-associated neurodegeneration
Source: Orphanet J Rare Dis. 2019 Jul 12;14:174. doi: 10.1186/s13023-019-1142-1 (PMC6625120; doi:10.1186/s13023-019-1142-1)
Supplement: Supplementary file 1 — Table S1. PKAN-specific medical history form. (DOCX 18 kb) [file 13023_2019_1142_MOESM1_ESM.docx]

**Table S1.** PKAN-specific medical history form

1. Date of assessment: __ __ - __ __ - 20__ __ 2. Patient age at time of interview: _______

dd mm yyyy

3. First problem you noticed that was related to PKAN (select all that apply):

□ Walking □ Speech □ Swallowing □ Writing □ Emotional or Behavioral Problems

□ Dystonia (select all that apply):

□ Mouth/Tongue □ Neck □ Hand □ Foot □ Back/Trunk

□ Other Dystonia:________________________________________________________________

□ Other Problems:_____________________________________________________________________

4. Age the first problem occurred (age of onset): ____ ____

5. Problem that led to the first doctor visit (select one only):

□ Same as #3 above □ Other: _____________________________________________________

6. Did you [the person with PKAN] ever get a brain scan (MRI)? □ No □ Yes

a. If “Yes”, age received first brain scan (MRI): ____ ____

b. If “Yes”, did the first brain scan lead to the PKAN diagnosis? □ No □ Yes

7. Did you [the person with PKAN] ever get genetically tested for PKAN? □ No □ Yes

a. If “Yes”, at what age were you [the person with PKAN] diagnosed with PKAN based on a genetic test? ____

b. If “No”, at what age were you [the person with PKAN] diagnosed with PKAN based on an MRI only? ______

8. About how many doctors did you [the person with PKAN] see before getting the diagnosis? ________

a. Were the doctors in your [the person with PKAN] community, or in a specialist center? (select all that apply):

□ Community Doctors □ Specialist Center Doctors

9. Treatments received (check any received to date):

□ Medication from a doctor □ Vitamins or other non-prescription/over the counter (OTC) treatments

□ Injections (e.g., botox/botulinum toxin, etc.) □ Therapies (physical, speech, occupational, etc.)

□ Surgery of any kind (select all that apply):

□ Placement of Deep Brain Stimulation device

□ Other Surgery: ___________________________________________________________

□ Other Treatments:____________________________________________________________

10. Approximate number of medical visits [the person with PKAN] in the last year (all types): ______

11. Approximate number of therapy (physical, speech, occupational, etc.) visits the person with PKAN had in the last year (all types): ______

| **DISEASE PROGRESSION** | |  |  |  |
| --- | --- | --- | --- | --- |
|  | **Medical Problems, Signs,**  **or Symptoms** | | **If “Yes”, the age first occurred** | **Notes** |
| **Problems With Walking** | |  |  |  |
| 12. | Unable to walk without help or support (other person, walker, cane, etc.)? | □ No  □ Yes | _____Years Old |  |
| 13. | Lost the ability to walk at all? | □ No  □ Yes | _____Years Old |  |
| **Problems With Speaking** | |  |  |  |
| 14. | Developed problems with speech and being understood? | □ No  □ Yes | _____Years Old |  |
| 15. | Lost the ability to speak at all? | □ No  □ Yes | _____Years Old |  |
| **Problems With Eating** | |  |  |  |
| 16. | Developed problems with eating or choking? | □ No  □ Yes | _____Years Old |  |
| 17. | Required placement of feeding tube? | □ No  □ Yes | _____Years Old |  |
| **Problems With Vision and Breathing** | | |  |  |
| 18. | Developed problems with vision? | □ No  □ Yes | _____Years Old |  |
| 19. | Required placement of tracheostomy tube for breathing? | □ No  □ Yes | _____Years Old |  |
| **General Problems** | |  |  |  |
| 20. | Has experienced a “dystonic storm” (sudden, severe dystonia attacks) according to the doctor? | □ No  □ Yes | _____Years Old |  |
| 21. | Problems in school of any kind related to PKAN (e.g. special needs, behavioral problems, etc.)? | □ No  □ Yes | _____Years Old |  |
| 22. | Required part-time caregiver? | □ No  □ Yes | _____Years Old |  |
| 23. | Required full-time caregiver? | □ No  □ Yes | _____Years Old |  |
